# A fresh view on the structure and twinning of owyheeite, a rod-polytype and twofold superstructure

**DOI:** 10.1107/S2052520623004523

**Published:** 2023-06-24

**Authors:** Berthold Stöger, Christian Göb, Dan Topa

**Affiliations:** a X-ray Centre, TU Wien, Getreidemarkt 9, 1060 Vienna, Austria; b Rigaku Europe SE, Hugenottenallee 167, 63263 Neu-Isenburg, Germany; c Mineralogisch-Petrographische Abteilung, Naturhistorisches Museum, Burgring 7, 1010 Vienna, Austria; University of Geneva, Switzerland

**Keywords:** mineral, superstructure, polytypism, twinning

## Abstract

Owyheeite [Cu_0.09 (1)_Ag_2.77 (4)_Pb_10.23 (4)_Sb_10.89 (5)_S_28.00 (5)_] crystallizes as a twofold superstructure with *P*2_1_/*n* symmetry and pseudo-orthorhombic metrics [*a* = 8.1882 (3) Å, *b* = 27.2641 (7) Å, *c* = 22.8679 (7) Å, β = 90.293 (3)°, *V* = 5105.0 (3) Å^3^, *Z* = 4]. Owyheeite is systematically twinned by reflection at (021) or equivalently (021). Twinning is explained by describing a simplified *Pmcn* archetype structure as polytype built of two kinds of rods, which contact via electron-pair micelles. A procedure of generating hypothetical polytypes by tiling space with partially overlapping *equivalent regions* is described.

## Abstract 2

Owyheeite crystallizes as a twofold superstructure. The systematic twinning is due to the rod-polytype character of the structure.

## 1. Introduction

Owyheeite is a mineral in the class of sulfosalts (Moëlo *et al.*, 2008 ▸) and was first described by Shannon (1921 ▸). Owing to systematic twinning and faint superstructure reflections, a full structure refinement has not been possible up to now. Laufek *et al.* (2007 ▸) refined the structure from synchrotron powder diffraction data in the space group *P*2_1_/*c* and the unit-cell parameters *a* = 4.1035 (1) Å, *b* = 27.3144 (3) Å, *c* = 22.9366 (3) Å, β = 90.359 (1)°. They observed, but did not account for, weak reflections with half-integer *h* values indicating a doubling of the *a*-axis to *a* ∼ 8.2 Å. The existence of the twofold superstructure had previously been reported by Robinson (1949 ▸). An order–disorder (OD) interpretation (Dornberger-Schiff & Grell-Niemann, 1961 ▸) of the systematic twinning has been given by Makovicky & Olsen (2015 ▸).

Using a modern diffractometer system with a hybrid photon counting detector and a highly collimated X-ray beam, we finally were able to refine the structure based on intensity data from a tiny needle of owyheeite. Here, a discussion of the twofold superstructure and an interpretation of the twinning based on partial pseudo-symmetry is given.

The material investigated by us is a portion of a specimen (2 × 3 mm) from Hector-Calumet mine, Elsa, Galena Hill, Mayo Mining District, Yukon, Canada given to one co-author (DT) by Mark Mauthner. Owyheeite is associated with boulangerite, zinkenite and sphalerite.

## 2. Experimental

### 2.1. Single crystal diffraction

Intensity data from a small needle of owyheeite were collected at room temperature on a Rigaku XtaLAB Synergy-S diffractometer system with finely collimated Mo *K*α radiation. Data were processed with the *CrysAlisPro* software (1.171.42.85*a*; Rigaku Oxford Diffraction, 2023 ▸). Two main domains were identified and frame data reduced to intensity values with overlap information (HKLF5-style reflection file). Intensities were scaled using the multi-scan approach and a Gaussian absorption correction was applied based on crystal faces.

The structure was solved with *SHELXT* (Sheldrick, 2015*a* ▸) and refined against |*F*|^2^ with *SHELXL* (Sheldrick, 2015*b* ▸). Data collection and refinement details are compiled in Table 1 ▸.

### 2.2. Electron microprobe analysis

**Figure 1:**
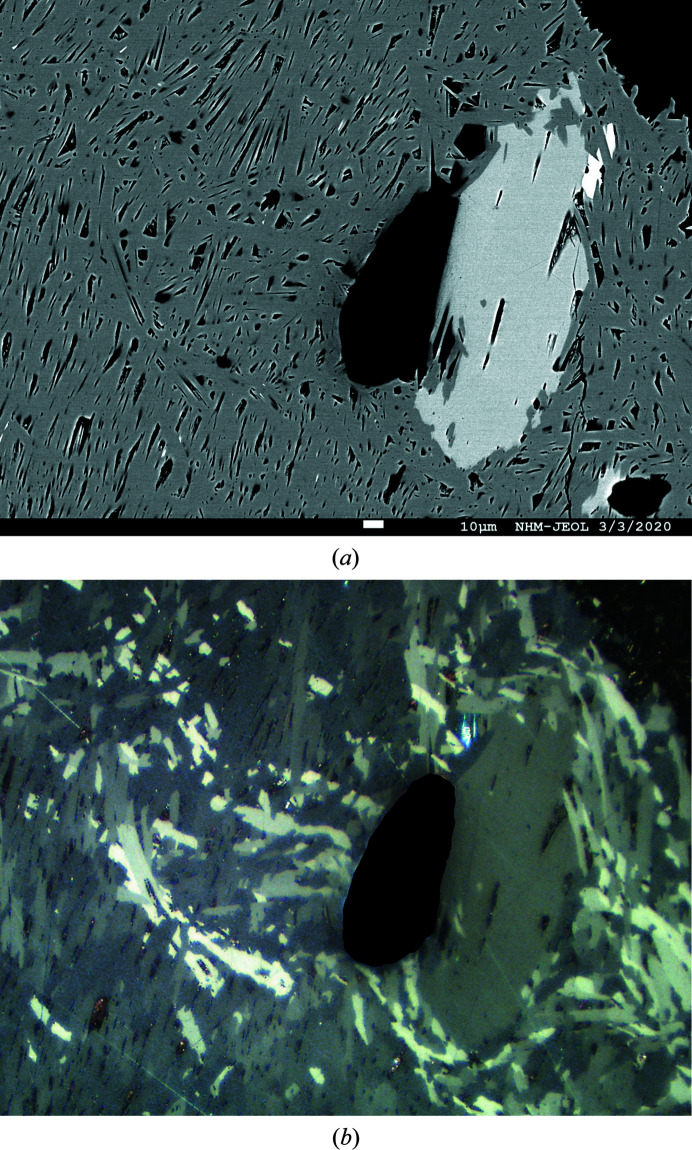
(*a*) Backscattered electron (BSE) image of owyheeite (gray) grains, associated with boulangerite (light gray), small inclusions of galena (white) and quartz (black). (*b*) Corresponding plane-polarized optical image showing dimensions and random orientation of owyheeite grains.

Ten points analyses on three grains were performed with a Jeol JXA 8530 F field emission electron microprobe in WDS mode at 25 kV, 20 nA, 2 µm beam diameter, using sulfide and metal standards and an online ZAF correction procedure (Fig. 1 ▸). The owyheeite empirical formula (52 apfu) is Cu_0.09 (1)_Ag_2.77 (4)_Pb_10.23 (4)_Sb_10.89 (5)_S_28.00 (5)_ with no serious variations among 10 individual analytical points and very close to the published empirical formula for owyheeite (Laufek *et al.*, 2007 ▸). The composition is reasonably close to the composition refined from single crystal data, Cu_0.20_Ag_2.80_Pb_9.77_S_28_Sb_11.23_. It must however be noted that the localization of the Cu position in the crystal structure is highly speculative (see below).

## 3. Results and discussion

### 3.1. Structural overview

Owyheeite is a twofold superstructure that can be derived from a *basic structure* with *P*2_1_/*c* symmetry and lattice parameter *a*_*b*_ = *a*/2 ∼ 4.1 Å. The corresponding basis vector will be called **a**_*b*_ = **a**/2. By ignoring the superstructure reflections, an *average structure* with unit-cell parameter *a*_*b*_ is obtained in structure refinements. Note that the basic and average structures are related yet subtly different [for details see, for example, van Smaalen (2007 ▸)].

**Figure 2:**
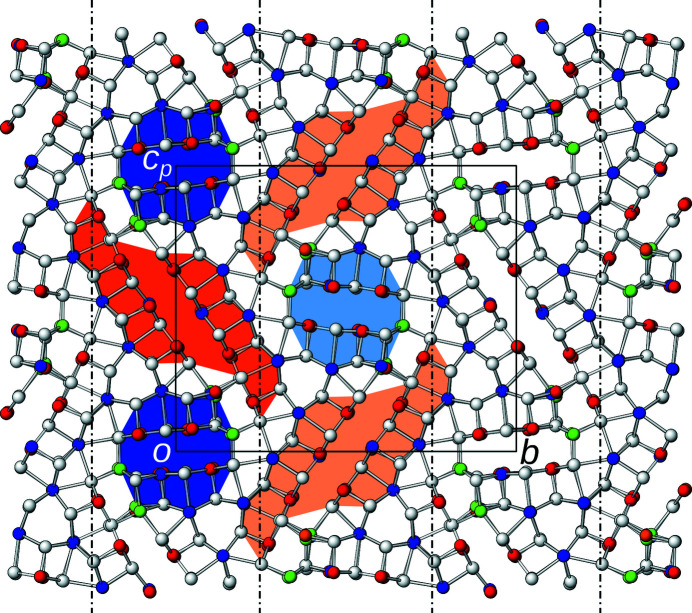
The crystal structure of owyheeite viewed down [100]. Atoms are represented by gray (S), blue (predominant Pb), red (predominant Sb), green (Ag) and orange (putative Cu) spheres of arbitrary radius. Blue and orange backgrounds mark the two kinds of crystal-chemical rods according to the interpretation of Laufek *et al.* (2007 ▸). Light colors of the background indicates a translational component of **a**/2. The *p*-subscript in *c*_*p*_ means that the axis is (slightly) out of plane, owing to the slightly obtuse angle β. The *n*_[010]_ glide reflection planes are indicated by the usual dash-dotted lines.

Since this average structure has been described in detail (Laufek *et al.*, 2007 ▸; Makovicky & Olsen, 2015 ▸), here only a short overview is given. The structure is monoclinic, but possesses pseudo-orthorhombic metrics [β = 90.292 (3)°]. As numerous other members of the sulfosalt family, owyheeite is a modular structure (Ferraris *et al.*, 2008 ▸). In the classical interpretation, it is described as being built of two kinds of rods extending along [100], marked by blue and orange backgrounds in Fig. 2 ▸. The rods can be considered as an intermediate between the PbS and SnS archetypes. The octahedral coordination is strongly distorted by formation of distinct long (orange background) and short (blue background) electron-pair micelles at the center of the rods, where predominantly Sb is located. The rods connect via predominant Pb positions with capped prismatic coordination and Ag positions with tetrahedral coordination. For a detailed description of the coordination polyhedra, see Laufek *et al.* (2007 ▸).

### 3.2. Superstructure

In the actual superstructure, *a* is doubled to *a* = 2*a*_*b*_ ∼ 8.2 Å, which means that the crystal-chemical rods described above can appear in two positions with respect to the *a*-axis (origin at *x* = 0 and ½, respectively). Rods related by the *c*_[010]_ glide reflection in the basic structure are additionally translated along **a**/2 as indicated by different shading of the blue and orange backgrounds in Fig. 2 ▸. Owing to the additional intrinsic translational component, the *c*_[010]_ glide reflection becomes an *n*_[010]_ glide reflection (indicated by the usual graphical symbol in Fig. 2 ▸) and the symmetry of the superstructure is accordingly *P*2_1_/*n*. The symmetry descent from the *P*2_1_/*c* basic structure to the *P*2_1_/*n* superstructure is of the *klassengleiche* type (retention of crystal class) and of index 2. More precisely, it is an *isomorphic* symmetry descent, since the space groups are of the same type.

Each site in the average structure splits into two sites in the superstructure (ignoring additional occupational disorder). Atoms corresponding to the same position in the average structure are labeled with the same number. Labels of atoms with *x*-coordinates in the 0–½ range end in an *A*, those in the ½–1 range with a *B*. Labels of S-sites start with S, of other sites with *M* (for metal).

Tables 2 ▸ and 3 ▸ list the distances of the atoms in the superstructure to those in the average structure. The latter were calculated by transforming into the *a*_*b*_ ∼ 4.1 Å cell and averaging the resulting coordinates. Sites with occupancies <½ were ignored for these considerations.

**Figure 3:**
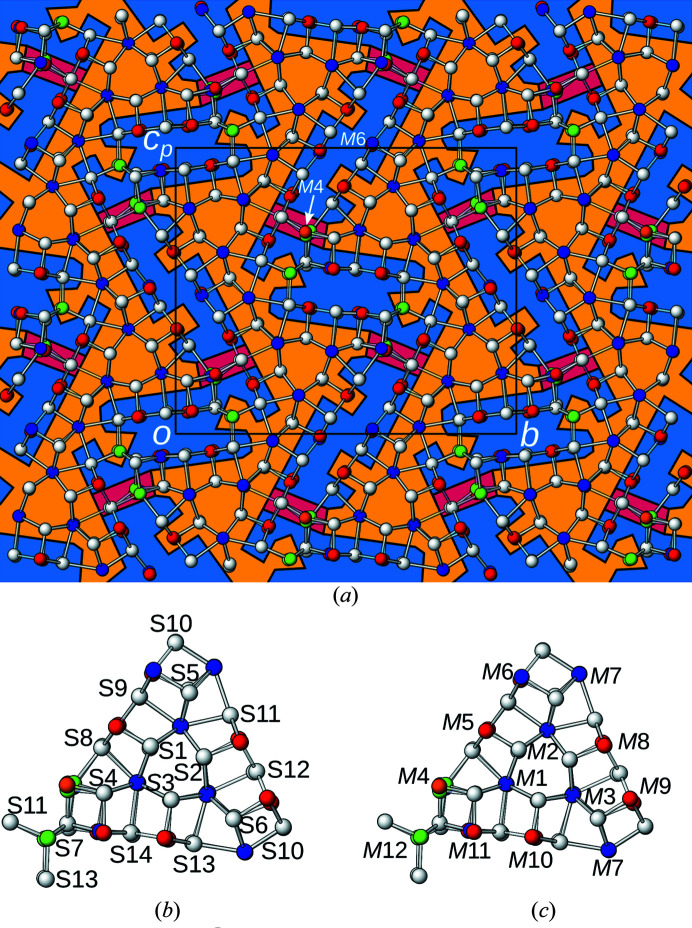
(*a*) The crystal structure of owyheeite viewed down [100]. Atoms are represented by gray (S), blue (predominant Pb), red (predominant Sb), green (Ag) and orange (putative Cu) spheres of arbitrary radius. Background according to modulation amplitude with respect to the average structure (yellow: <0.10 Å, blue: 0.10–0.24 Å, red: >0.24 Å). Numbering schemes of the (*b*) S- and (*c*) *M*-sites. Slightly more than an asymmetric unit is shown for better orientation.

The modulation amplitude is color-coded in Fig. 3 ▸(*a*) and the atom-naming scheme given in Figs. 3 ▸(*b*) and 3 ▸(*c*). The largest modulation amplitudes (>0.24 Å from the averaged position) are observed for the S12*A*/S12*B* and *M*4*A*/*M*4*B* sites (red background). Atoms which show less than 0.10 Å modulation amplitude are marked by a yellow background and atoms with an intermediate modulation (0.10–0.24 Å) by a blue background.

The part of the structure that shows less modulation is the ‘surface’ of the crystal-chemical rods described in the previous section (§3.1), whereas the ‘center’ of the rods feature distinctly more pronounced modulation. This will lead to an alternative rod description below.

Besides positional modulation, formation of a superstructure can also affect occupancies. These are compiled in the two rightmost columns of Table 3 ▸ (no occupational modulation occurs for S atoms). The structure features practically no occupational modulation with two exceptions. Firstly, the *M*4*A*/*M*4*B* sites mentioned previously and secondly the *M*6*A*/*M*6*B* sites, which are Sb/Pb mixed positions at the midpoint of the ‘long’ electron-pair micelles [see Fig. 3 ▸(*a*) for position]. The *M*6*A* position is practically pure (98%) Sb as expected for atoms bordering the micelles. However, perhaps surprisingly, the *M*6*B* position is majorly (73%) Pb.

**Figure 4:**
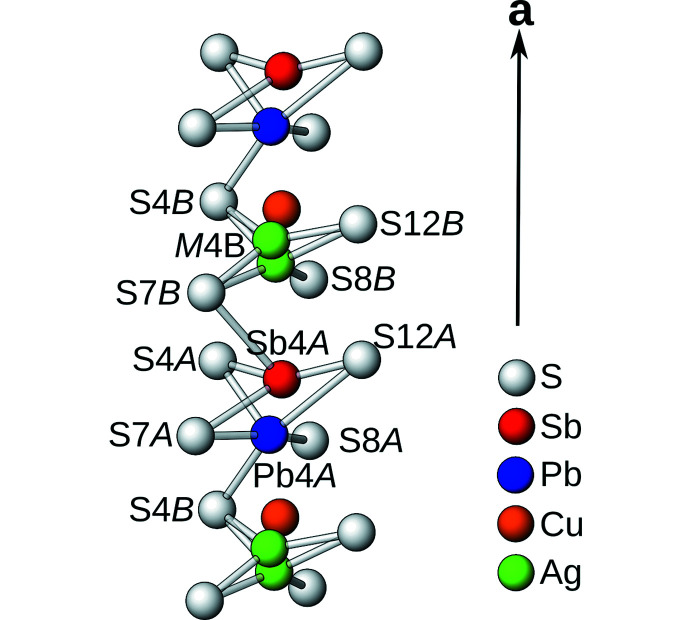
Column of disordered *M*4*A*/*M*4*B* sites with coordinating S atoms.

The *M*4*A*/*M*4*B* site is unique, as it is heavily positionally modulated *and* occupationally disordered. The *M*4*A*/*M*4*B* atoms and the coordinating S atoms are depicted in Fig. 4 ▸. The *M*4*A* site was refined as a Pb (76%) and an Sb (24%) position. Considering Pb⋯S contacts up to 3.6 Å, the Pb4*A* position is coordinated by a square pyramid [2.572 (8)–3.432 (6) Å]. A further longer contact to S12*B* [3.664 (6) Å] completes the strongly irregular octahedral coordination. The minor Sb4*A* sites features four short contacts [2.386 (10)–3.362 (12) Å] and a longer Sb4*A*⋯S8*A* contact [3.568 (10) Å]. The irregular polyhedron can be derived from a trigonal bipyramid.

On the *M*4*B* site three distinct electron-density peaks were observed, which were assigned to two Ag atoms and a Cu atom. However, assignment of the latter is purely speculative and based on the composition determined from microprobe analysis and very short *M*—S distances, which might however be virtual. The major position (72% Ag) is coordinated by a flattened tetrahedron [2.542 (7)–2.844 (7) Å]. The coordination of the minor Ag position (10%) appears trigonally planar, though local changes of the coordinating S atoms may not be apparent from X-ray diffraction. Likewise, the putative Cu atom (18% occupancy) features two very short apparent contacts [2.26 (3) and 2.34 (3) Å], which may however not be realized.

**Figure 5:**
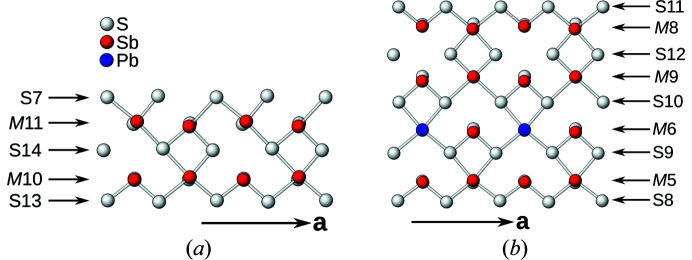
‘Surface’ of the (*a*) short (blue in Fig. 2 ▸) and (*b*) long (orange in Fig. 2 ▸) electron-pair micelles. *M*⋯S contacts are indicated up to 3.0 Å. Minor positions are omitted for clarity.

The pronounced modulation of the electron pair micelles is reflected by a distinct asymmetry of the Sb coordination polyhedra (Fig. 5 ▸). Ignoring contacts across the micelles, the coordination can be considered as distorted square pyramidal, or ψ^1^-octahedral when including the electron pairs. When (arbitrarily) choosing 3.0 Å as the limit between short and intermediate length Sb—S bonds, the modulation forms characteristic patterns. The contact to the apex of the pyramid (the S atoms obstructed in Fig. 5 ▸ by the *M* atoms) is always short, practically always less than 2.5 Å. Only for the *M*6*B* position, which however is mostly Pb, a longer distance of 2.641 (6) Å is observed. In the short micelles [Fig. 5 ▸(*a*)], one row of Sb atoms features alternately 5 (Sb10*A*) and 3+2 (Sb10*B*) coordination. In contrast, the second row (Sb11*A*/Sb11*B*) features only 4+1 coordination, whereby the intermediate length contact alternates between two positions. In the long micelles [Fig. 5 ▸(*b*)] only an alternating sequence of 5 and 3+2 coordinations are realized, whereby adjacent rows are offset by **a**/2. In the *M*6*A*/*M*6*B* row, the position of the 5 coordinated *M* atom is substituted by ∼73% Pb (see also Table 3 ▸).

### 3.3. Twinning and polytypism

#### 3.3.1. OD Theory

Owyheeite is twinned by reflection at the (021) or equivalently 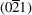 planes. Makovicky & Olsen (2015 ▸) have presented an OD interpretation (Dornberger-Schiff & Grell-Niemann, 1961 ▸) of the twinning of owyheeite. In such an OD interpretation, partial symmetry, *i.e.* symmetry that is valid only for a distinct modules, usually diperiodic layers, leads to an ambiguity in the arrangement of the modules. It is only required that adjacent modules form geometrically equivalent pairs, meaning that all possible arrangements are locally equivalent. Thus, OD theory can be considered as a generalization of the concept of crystalline matter to short-range order.

The symmetry of an OD structure is described by a *groupoid* of *partial operations* (POs) mapping a module onto itself or onto a different module. These OD groupoids are categorized into OD groupoid families, which correspond to the 230 space group types in classical crystal structures. The linear parts of POs that are not valid for the whole structure often appear as twin operations. Thus, OD groupoid families offer an unified view on a family of polytypes and the potential twin operations or other stacking faults (such as formation of antiphase domains) of its members.

Not all of the POs correspond to full symmetry operations and therefore OD structures typically feature *desymmetrization* (Ďurovič, 1979 ▸) with respect to an idealized model.

Application of the OD theory serves two distinct purposes. Firstly, it allows the concise description and prediction of various forms of stacking disorders. Secondly, on a higher level, it allows for a classification of whole families of polytypic structures. The second may require a higher degree of idealization.

#### 3.3.2. A simplified structure

To keep the OD description simple, we will apply the OD theory to a simplified *archetype* structure. An application of the OD theory to the owyheeite superstructure would result in numerous different putative twin composition planes, which cannot be differentiated by routine diffraction methods. In fact, Fig. 3 ▸ shows that parts of the structure with large modulation are separated by parts with negligible modulation. Since the former feature less translation symmetry, application of the OD theory would predict alternative possible placements of the former with respect to the latter. However, so far there is no evidence of such an ambiguity. Moreover, as we will show below, alternative polytypes possess electron-pair micelles of a different shape, which might possess a different modulation. Instead, here we attempt to develop a model that is as simple as possible, yet complex enough to explain the observed twinning and to provide a building principle that can be applied to related structures with different modulations.

Firstly, we will only consider the basic *P*2_1_/*c* structure with unit-cell parameter *a*_*b*_ = *a*/2 ∼ 4.1 Å. Moreover, we will assume reflection symmetry at planes *x* = ½. The resulting, distinctly simpler, structure has orthorhombic *Pmcn* symmetry. The full symbol *P*2_1_/*m*2_1_/*c*2_1_/*n* emphasizes the supergroup relation with *P*2_1_/*c*. This pseudo-symmetry has already been noted by Makovicky & Olsen (2015 ▸) ▸. Even though the upcoming discussion will pertain to the simplified *Pmcn* archetype structure, some figures will show the actual structure to give an impression of the deviation from this idealized symmetry.

#### 3.3.3. A structure of rods

The OD interpretation of Makovicky & Olsen (2015 ▸) in terms of OD layers is not entirely satisfying, because the OD groupoids do not contain all operations of the actual polytype and introduction of ‘accidental’ symmetry is required. An analogous phenomenon is observed for the close-packing of spheres, when described as an OD structure of hexagonal layers. For the 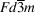 cubic closest packing, additional ‘accidental’ symmetry operations, namely threefold rotations with an axis inclined to the layer planes, are not contained in the OD groupoid and appear ‘out of nowhere’. The close-packing of spheres is therefore a poor example of an OD structure.

This shortcoming in the OD description of owyheeite is due to the fact that the twin plane is at an oblique angle to the monoclinic axis of owyheeite. The OD layers were chosen by Makovicky & Olsen (2015 ▸) as extending parallel to (021). The symmetry operations in the [010] direction of the actual structure cannot be contained in the OD groupoid because they would map a layer onto a layer extending parallel to 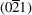 , which intersects the original layer. In fact, these 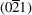 layers are a symmetrically equivalent description to the (021) layers. Ultimately, a unified description can only be obtained by modeling the structure in terms of periodic rods extending along [100].

As has already been noted by Makovicky & Olsen (2015 ▸), the twin elements form an angle close to 120° with the symmetry elements of the structure. Here, we will assume ideal 120° angles. Deviations therefrom are due to desymmetrization of the actual polytypes.

**Figure 6:**
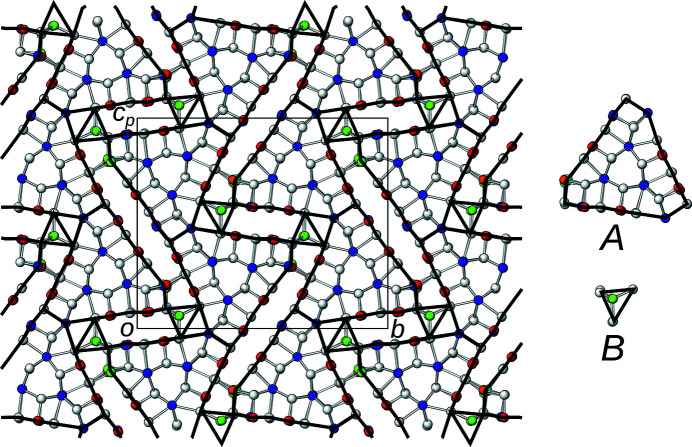
The crystal structure of owyheeite viewed down [100]. Atoms are represented by gray (S), blue (predominant Pb), red (predominant Sb), green (Ag) and orange (putative Cu) spheres of arbitrary radius. Lines mark the outline of the *A*- and *B*-rods.

The first step in an OD interpretation is identifying the modules. Reflection planes encompassing a 120° angle require a threefold rotational symmetry along the plane intersections. In the least modulated parts of the structure (yellow background in Fig. 3 ▸), there are suggestive rods of Pb atoms coordinated by bicapped trigonal prisms located around a threefold pseudo-rotation axis, as has already been noted by Laufek *et al.* (2007 ▸). Extending outward from this structural element the atoms that still follow this symmetry are identified. Ultimately, the truncated triangular rods shown in Fig. 6 ▸ are obtained, which will be designated as *A*-rods. The threefold rotation and the *x* = ½ reflection plane combine to give 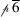 rod symmetry. Similar trigonal modules have been identified in numerous other sulfosalt minerals (Makovicky, 1985 ▸).

**Figure 7:**
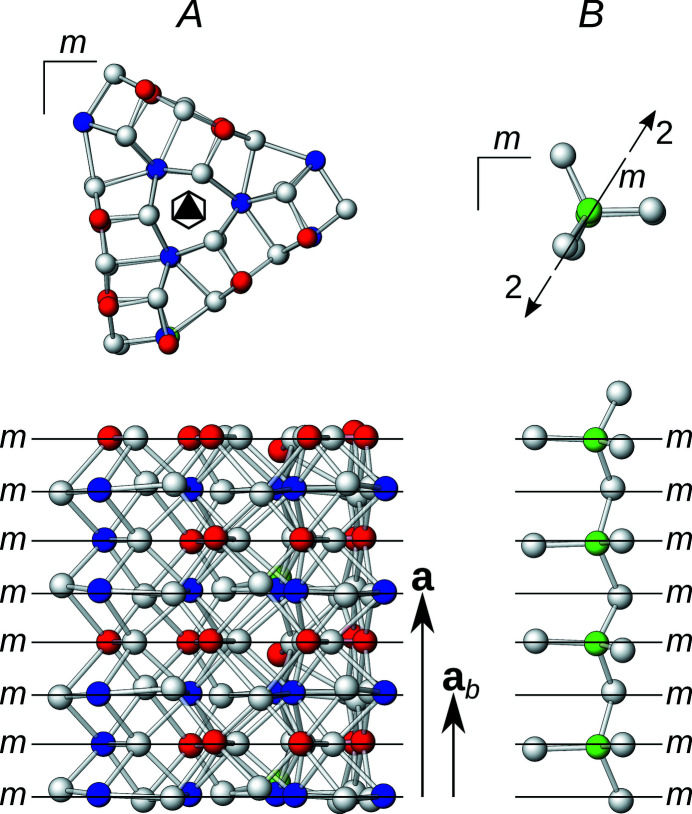
The (left) *A*- and (right) *B*-rods viewed (top) along and (bottom) perpendicular to the rod axis. Atoms are represented by gray (S), blue (predominant Pb), red (predominant Sb), green (Ag) and orange (putative Cu) spheres of arbitrary radius. (Pseudo-)symmetry elements are indicated by the usual graphical symbols.

By identifying the *A*-rods, all atoms with the exception of the Ag13 atoms are located either in or at the boundary of the rods. Thus, Ag13 and the coordinating S atoms form a second kind of rod, called *B*-rods, with 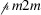 symmetry (Fig. 6 ▸). The symmetry of both kinds of rods is shown in Fig. 7 ▸.

#### 3.3.4. Maximum equivalent regions

The partitioning of the structure into *A*- and *B*-rods is clearly not OD in the classical sense, which would mean that equivalent sides of an *A*-rod connect to adjacent rods in geometrically equivalent ways. However, the *A*-rods possess three equivalent ‘short’ sides, two of which connect to other *A*-rods via a shared face and the third to a *B*-rod via an edge.

**Figure 8:**
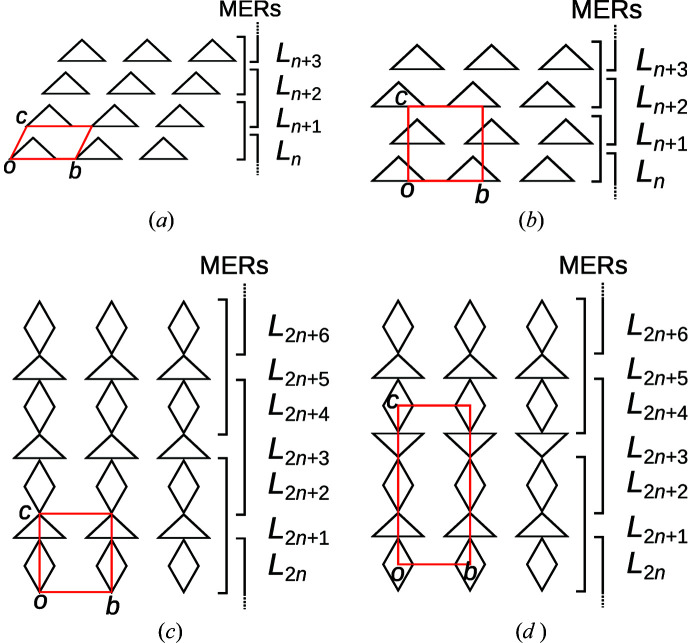
(*a*,*b*) Schematic representation of two members of an OD family of layer structures with MERs of two layers width. (*c*,*d*) Schematic representation of two members of a family of non-OD layer structures, where the polytypes are nevertheless fully covered by MERs. Odd-numbered layers can appear in one of two orientations.

Nevertheless, the structure can still be considered to have OD character. In fact, the crucial part of an OD interpretation is that all polytypes of a family are locally equivalent. In classical OD structures, every pair of adjacent modules is part of a *maximum equivalent region* (MER), which is the largest region that is equivalent in all polytypes of the family (Grell, 1984 ▸). Since the structure is fully covered by MERs consisting of at least two modules, every point of a polytype is located at least one module deep in an MER [see Figs. 8 ▸(*a*) and 8 ▸(*b*)].

However, such a situation can also be constructed with modules contacting in non-equivalent ways as shown in Fig. 8 ▸(*b*). We have named these kinds of structures *non-classical* OD structures (Stöger & Weil, 2013 ▸).

**Figure 9:**
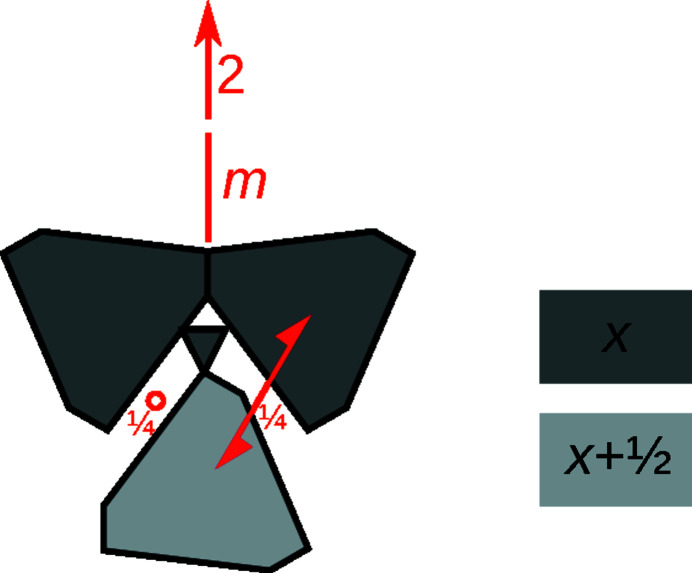
Schematic representation of a MER in the archetype structure of owyheeite consisting of three *A*-rods and one *B*-rod viewed down [100]. Different levels of gray indicate a translation of **a**_*b*_/2. POs of particular interest (see text) are indicated in red.

In owyheeite, one can also identify such an MER composed of three *A*-rods and one *B*-rod, as schematized in Fig. 9 ▸.

To build the structure from MERs, one has to know their partial symmetry. The POs mapping a rod onto itself, called λ-POs in OD theory, correspond to the rod groups of the *A*- and *B*-rods as described above. The POs mapping two distinct rods are called σ-POs. Since rod groups are infinite, there is also an infinite number of σ-POs relating two given rods of the same type. Since all rods possess the same translation group 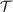 it is convenient to consider σ-POs only up to translation. Then two *A*-rods are related by 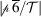 = 6 and two *B*-rods by 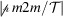 = 4 classes of σ-POs.

The two adjacent *A*-rods of the MER sharing a face are mapped by a reflection and a twofold rotation as in indicated in Fig. 9 ▸ (top of figure). The linear parts of these two operations remain as twin law. Their symmetry elements (reflection plane and rotation axis) coincide with those of the *B*-rod. This is an important fact, as it means that the *B*-rod can only be placed in one way with respect to the *A*-rods. The remaining four classes of σ-POs relating adjacent *A*-rods are glide reflections and screw rotations, which are not indicated in Fig. 9 ▸ for clarity.

The top-left and bottom *A*-rods in Fig. 9 ▸ are mapped by an inversion, which remains as a total operation of the owyheeite structure. It is located at *x* = ¼ which means that the origins of the rods are shifted along **a**_*b*_/2, which is indicated by a different shade of gray in Fig. 9 ▸. The other classes of σ-POs are two- and sixfold screw rotations and threefold rotoinversions.

The top-right and bottom *A*-rods in Fig. 9 ▸ are mapped by twofold screw rotations and glide reflections. The screw axis indicated in Fig. 9 ▸ is a symmetry operation of the archetype structure.

#### 3.3.5. Polytypes

**Figure 10:**
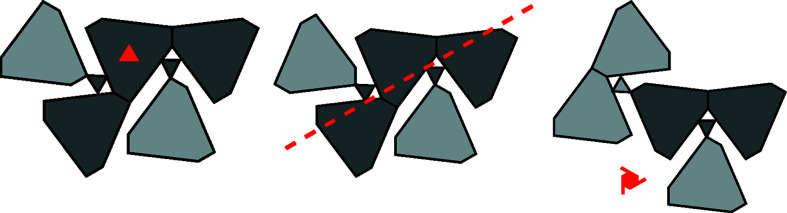
Three geometrically non-equivalent ways of extending the MER of Fig. 9 ▸ with another MER, such that they share the top-left *A*-rod of Fig. 9 ▸. A representative operation mapping the first MER to the second is indicated by the graphical symbol of the corresponding symmetry element (note that graphical symbols are defined for full symmetry elements, not single symmetry operations).

In analogy to OD structures of layers, the whole polytype can be grown by applying the λ- or σ-POs described in the previous section to the MER. Since the POs are valid for individual *A*-rods, but not the whole MER, non-equivalent fragments are thus obtained. For example, Fig. 10 ▸ shows three non-equivalent ways of applying λ- or σ-POs to the MER shown in Fig. 9 ▸. All three cases are partial overlays of two MERs sharing a common *A*-rod.

**Figure 11:**
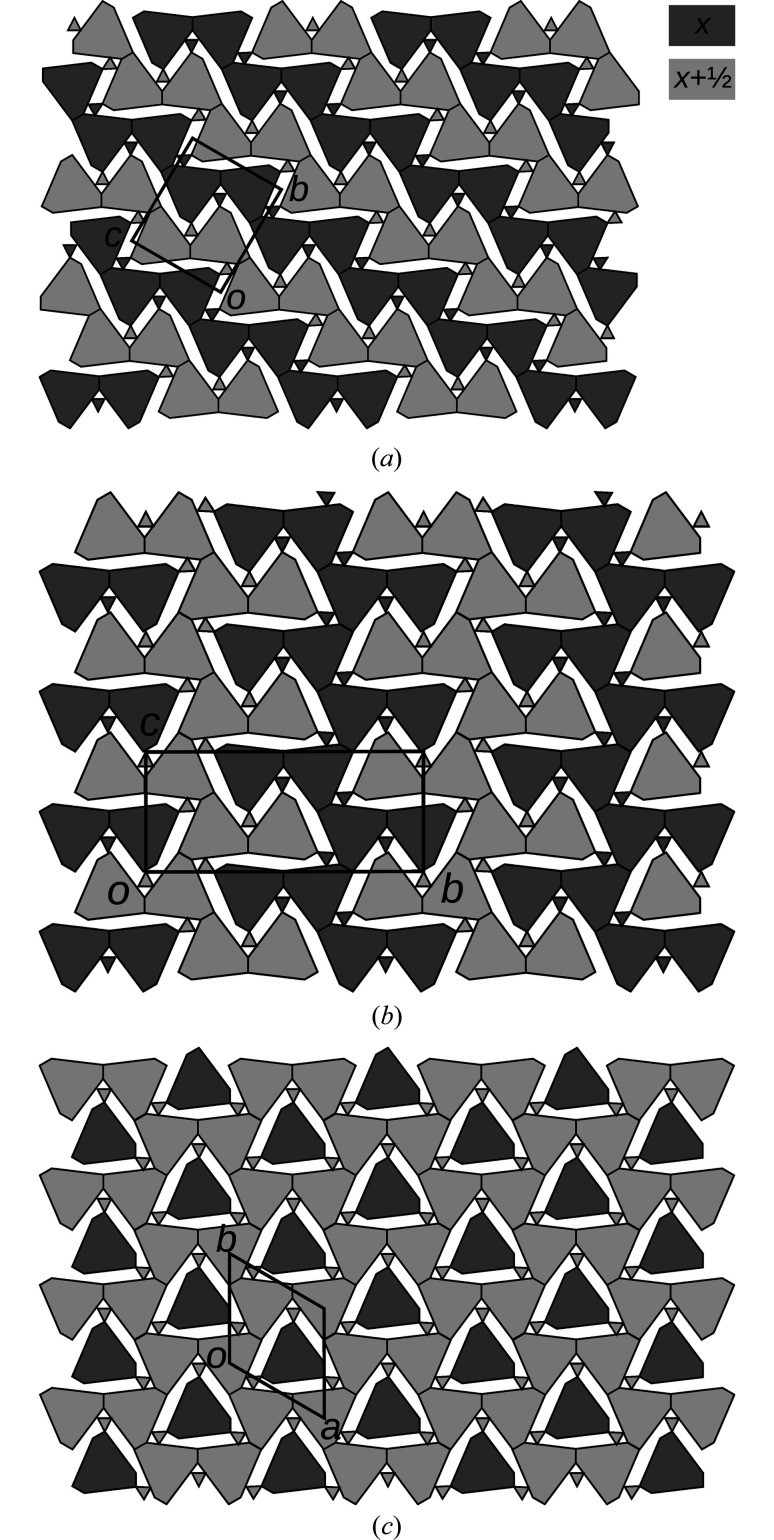
Schematic representation of three distinct polytypes obtained by combining MERs: (*a*) *Pmcn* archetype owyheeite, (*b*) isomorphic *Pmcn* symmetry, (*c*) a putative 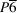 polytype. Light colors indicate a translation along **a**/2.

By repeated extension of the newly generated fragment with MERs, full polytypes can be grown. Here, a significantly increased complexity of rod polytypes manifests itself, as not all combinations of MERs lead to valid structures. Thus, whereas enumerating all layer polytypes is generally trivial, doing the same for rod polytypes may be a challenging combinatorial problem. Examples of possible polytypes are schematized in Fig. 11 ▸. Note that every *A*-rod belongs to three MERs.

Fig. 11 ▸(*a*) shows the *Pmcn* archetype owyheeite structure. Fig. 11 ▸(*b*) gives an alternative polytype with isomorphic *Pmcn* symmetry, but a different shape of the unit cell. These two polytypes are constructed of equivalent layers (extending horizontally in Fig. 11 ▸). They are members of the OD family of layers suggested by Makovicky & Olsen (2015 ▸) ▸, if one ignores the issue of ‘accidental’ symmetry. An example of a putative polytype that cannot be derived from the layer model is schematized in Fig. 11 ▸(*c*). It has hexagonal 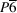 symmetry and is the simplest representative of a whole family of polytypes, since every *A*-rod with a dark shade in Fig. 11 ▸(*c*) can appear in one of two orientations.

**Figure 12:**
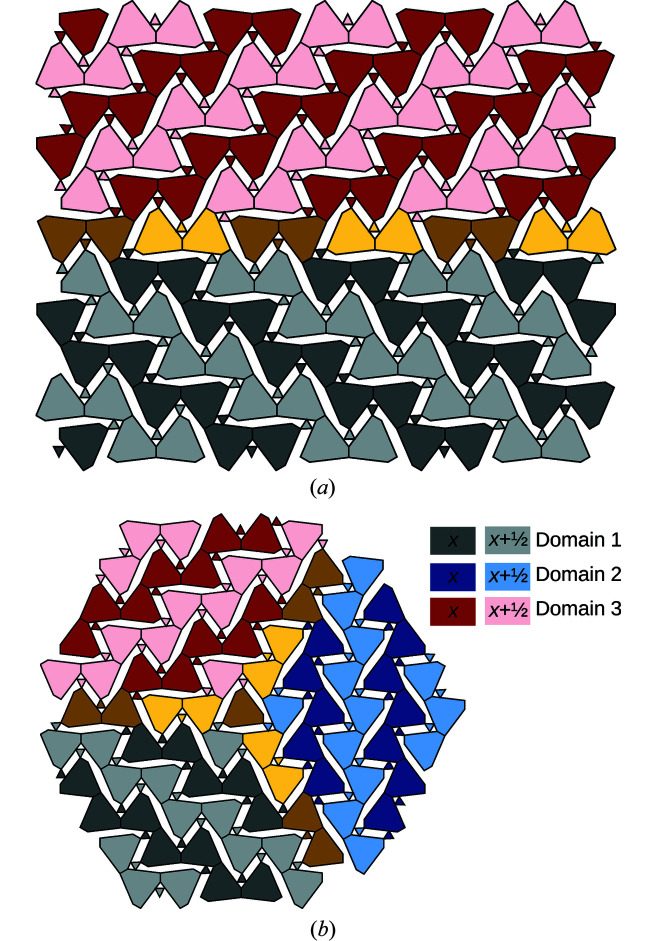
Schematic representation of (*a*) a twin and (*b*) a trill (a twin with three orientation states) of the owyheeite archetype structure. Gray, red and blue represent different twin domains. Parts of the structure that can be attributed to two or three domains are colored in yellow. Light colors indicate a translation along **a**/2.

Twins of the owyheeite archetype can be formed as shown in Fig. 12 ▸(*a*), where a fragment of the polytype of Fig. 11 ▸(*b*) is located between both twin domains. The orientations of the twin individuals are related by the linear part of a reflection σ-PO (see Fig. 9 ▸). Owing to the threefold symmetry of the *A*-rods, the composition plane can also occur in other directions. The possible twin orientation states of the owyheeite archetype structure can be derived by coset decomposition of the point group of the structure in the point group of the groupoid of all POs. These are the point groups generated by the linear parts of all symmetry operations and all POs, respectively. Thus, there are |6/*mmm*|/|*mmm*| = 3 possible orientation states, with a threefold axis as twin element.

For example, Fig. 12 ▸(*b*) gives an example of a trill (twin with three orientation states) meeting in a single rod. As before, the interface between two domains are sheet-like fragments of the polytype in Fig. 11 ▸(*b*). Where all three domains meet, a rod-like fragment of the Fig. 11 ▸(*c*) polytype is realized. As noted by Makovicky & Olsen (2015 ▸), such a trill might not be stable in actual owyheeite owing to strong metric distortions of the bulk domains.

#### 3.3.6. Electron-pair micelles

The schemes in Figs. 11 ▸ and 12 ▸ feature white areas, which represent regions of non-interaction between the *A*- and *B*-rods that have been ignored so far. In general, the possibility of empty space between rods is a further complication compared to layer polytypes and may appear for example in zeolites or metal–organic frameworks (MOFs). For owyheeite, these regions are not empty, but correspond to the electron-pair micelles discussed in §3.1. The model above assumed that these are not structure directing, *i.e.* the rods can contact in arbitrary ways. Evidently, this is a strong idealization, as there will be interactions across the electron-pair micelles.

**Figure 13:**
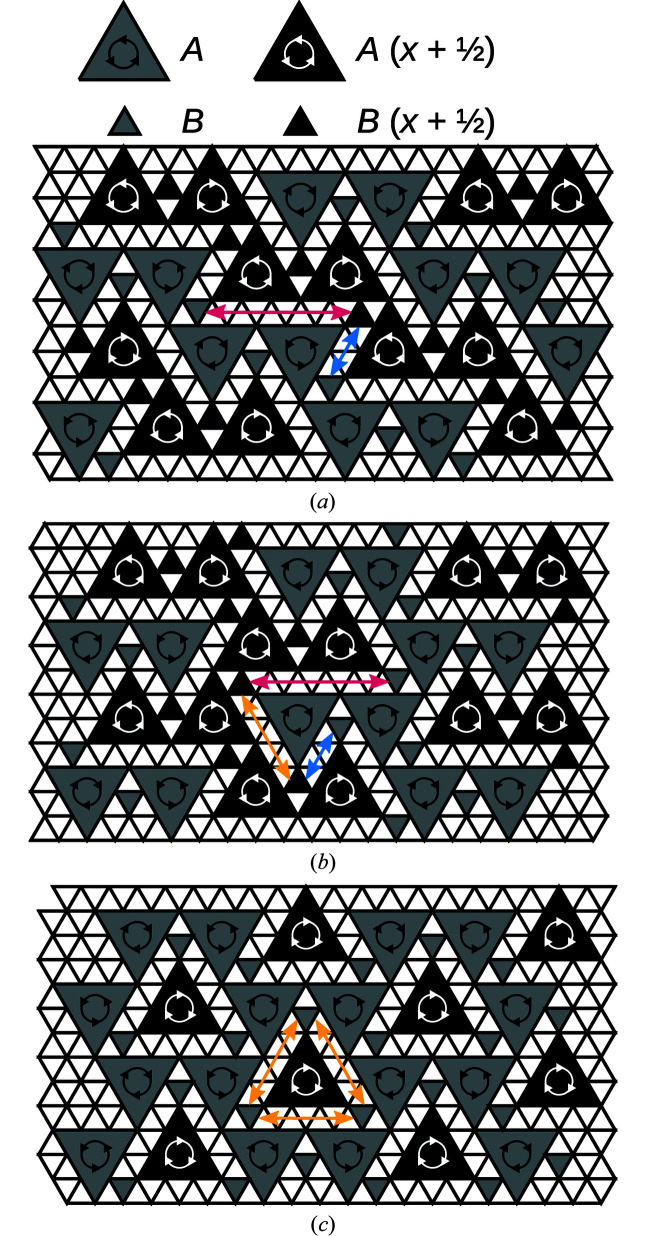
Schematic representation of the polytypes from Fig. 11 ▸ by triangles placed on a triangular net. The orientation of the *A*-rods is given by circular arrows. Different kinds of electron-pair micelles are indicated by colored arrows.

If the micelles are considered as individual modules, the polytypes cease having OD character. To show this, we increase the level of abstraction and represent the arrangement of the *A*- and *B*-rods by oriented equilateral triangles, whereby the *A*-rod triangles have a tripled side length compared to the *B*-rod triangles. The triangles can then be placed on a trigonal net as shown in Fig. 13 ▸. The electron-pair micelles are the remaining space, *i.e.* small unshaded triangles. In the owyheeite archetype [Fig. 13 ▸(*a*)], two kinds of micelles exist, a long and a short one marked by red and blue arrows, respectively. These correspond to the short and long rods marked in Fig. 2 ▸. In the polytype schematized in Fig. 13 ▸(*b*) [corresponding to Fig. 11 ▸(*b*)], a third intermediate-length micelle exists, marked by a yellow arrow. Such micelles are therefore expected to exist at the interface between two twin domains. The micelle differs from the previous ones in that the adjacent *B*-rods are at the same *x*-coordinates (note the shading of the *B*-rods at the long end of the micelles). The hypothetical *P*6/*m* and related polytypes of Fig. 11 ▸(*c*) only possess micelles of this kind [see Fig. 13 ▸(*c*)].

Thus, when considering the electron-pair micelles as building blocks, the structure family does not have OD character, since the *A*- and *B*-rods connect to different micelles. Ultimately, one might even consider the structures as not being polytypes, but rather members of a *merotype* family (Ferraris *et al.*, 2008 ▸), since they may contain *different types* of electron-pair micelles. Despite the varying spatial distribution of *A*- and *B*-rods, in all polytypes there are as many *A*-rods as *B*-rods.

Note that the modulation of atoms in the actual owyheeite superstructure is distinctly more pronounced for the electron-pair micelles and the strongest modulation (*M*4*A*/*M*4*B* position) is precisely located at the transition from short to long micelles (see Fig. 3 ▸). Standard diffraction methods do not allow a structural characterization of the twin interface. Therefore, the exact nature of the intermediate electron-pair micelle is unknown. It might feature a different modulation period or a different chemistry, which substantiates the choice of basing the model on a simplified archetype structure. The latter could mean the twin interface is allochemical.

#### 3.3.7. Relationship to the OD interpretation in terms of layers

**Figure 14:**
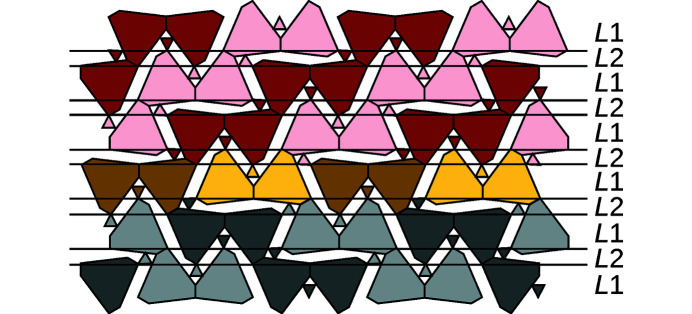
The twin interface schematized in Fig. 12 ▸(*a*) interpreted in terms of an OD structure of layers. Horizontal lines indicate the layer boundaries. Layer types are indicated to the right. Color codes as in Fig. 12 ▸.

The rod interpretation given above was derived by combining the OD interpretation of Makovicky & Olsen (2015 ▸) with the space group symmetry of the archetype structure. The model should therefore reduce to the original OD model if only a single twin-plane is considered. This is in fact the case, as schematized in Fig. 14 ▸. Makovicky & Olsen (2015 ▸) proposed an alternating sequence of two kinds of OD layers. Thick layers, which we designate as *L*1, feature reflection planes perpendicular to the layer planes and which correspond to the reflection planes indicated in Fig. 9 ▸. The thinner *L*2-layers do not possess such a symmetry. Note that application of the reflection plane of an *L*1-layer onto the adjacent *L*2-layer changes the connectivity of the *A*- and *B*-rods and generates an alternative electron-pair micelle at the twin interface that does not exist in the twin individuals as described in the previous section.

#### 3.3.8. An alternative three-rod model

**Figure 15:**
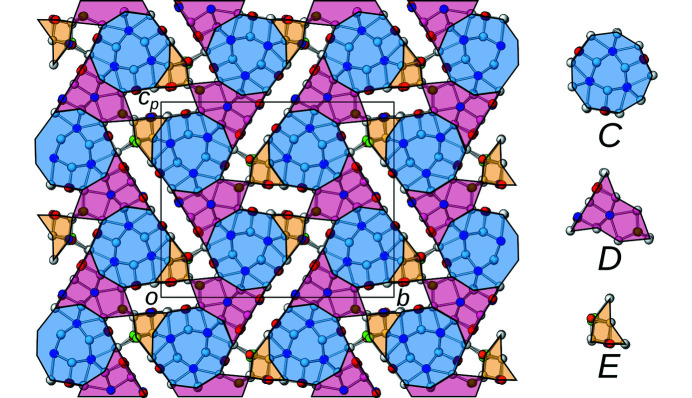
The crystal structure of owyheeite viewed down [100]. Atoms are represented by gray (S), blue (predominant Pb), red (predominant Sb), green (Ag) and orange (putative Cu) spheres of arbitrary radius. Background colors according to a three-rod interpretation with less idealization.

A characteristic feature of the owyheeite structure is the *M*4 column (Fig. 4 ▸), which is located at the ‘corner’ of the *A*-rods close to the contact point of the *B*-rods. In the actual polytype [Fig. 11 ▸(*a*)], there is one such *M*4 column per *A*-rod. In the polytypes of Figs. 11 ▸(*b*) and 11 ▸(*c*) one might also expect none, two or three *M*4 columns, according to the number of connected *B*-rods. In a strict sense, these different kinds *A*-rods could therefore be considered as non-equivalent. When allowing for less idealization the corners of the *A*-rods have to be omitted, resulting in smaller *C*-rods, marked by a blue background in Fig. 15 ▸. Two of the three corners of the *A*-rods are then attributed to the *B*-rods, giving a new *D*-rod (red in Fig. 15 ▸). The third corner, which features the *M4* column, becomes a third kind of rod, designated as *E* (yellow in Fig. 15 ▸). The resulting three-rod model is equivalent to the two-rod model with MERs being built of three *C*-rods, one *D*-rod and one *E*-rod. It is a common phenomenon in OD structures that varying levels of idealization lead to equivalent models with a different number of kinds of layers. The interface between modules can be considered as its own module. Here, we focused on a more general model with fewer modules to emphasize the general building principle of the structure family and not the actual chemistry of this particular member.

## 4. Conclusion and outlook

A description of modular structures is incomplete without consideration of partial symmetry. Only a rod-model allows for a comprehensive symmetry description from which the symmetry of the owyheeite (archetype) structure *and* the twin operations can be derived. The model also provides a deeper understanding of the nature of the twin domain, notably the different electron-pair micelles located at the twin interface. A theory of polytypes of rods is significantly harder to achieve than for layers and therefore only a little work has been performed in this direction.

A second point of note is that the OD character of a structure is perhaps not ideally expressed by decomposing the structure into disjoint modules. Instead, we suggest considering MERs as the basic building blocks of the structure. The operations relating MERs are likewise partial operations that form groupoids. In this case however, the domains and codomains of the operations do partially overlap. These types of groupoids will allow for a finer classification of modular structures.

Finally, the rod interpretation given here has been derived indirectly from twinning. Alternative arrangements are realized at the twin interface. The ultimate proof of the interpretation’s validity will, however, require finding macroscopic polytypes. 

## Supplementary Material

Crystal structure: contains datablock(s) I. DOI: 10.1107/S2052520623004523/ra5131sup1.cif

Structure factors: contains datablock(s) I. DOI: 10.1107/S2052520623004523/ra5131Isup2.hkl

CCDC reference: 2264833

## Figures and Tables

**Table 1:** Experimental details for owyheeite

Crystal data	
Chemical formula	Ag_2.80_Cu_0.20_Pb_9.77_S_28_Sb_11.23_
*M*_r_	4604.25
Crystal system, space group	Monoclinic, *P*2_1_/*n*
Temperature (K)	298
*a*, *b*, *c* (Å)	8.1882 (3), 27.2641 (7), 22.8679 (7)
β (°)	90.283 (3)
*V* (Å^3^)	5105.0 (3)
*Z*	4
Radiation type	Mo *K*α
μ (mm^−1^)	40.20
Crystal size (mm)	0.06 × 0.02 × 0.01

Data collection	
Diffractometer	Rigaku XtaLAB Synergy, Dualflex, HyPix-6000 HE
Absorption correction	Multiscan (*CrysAlisPRO*; Rigaku Oxford Diffraction, 2023 ▸). Numerical absorption correction based on Gaussian integration over a multifaceted crystal model. Empirical absorption correction using spherical harmonics, implemented in SCALE3 ABSPACK scaling algorithm.
*T*_min_, *T*_max_	0.167, 0.800
No. of measured, independent and observed [*I* > 2σ(*I*)] reflections	40130, 22895, 15639
*R*_int_	0.044
(sin θ/λ)_max_ (Å^−1^)	0.714

Refinement	
*R*[*F* ^2^> 2σ(*F* ^2^)], *wR*(*F* ^2^), *S*	0.066, 0.146, 1.02
No. of reflections	22895
No. of parameters	495
No. of restraints	1
	*w* = 1/[σ^2^(*F*_o_^2^) + (0.0506*P*)^2^ + 282.6104*P*] where *P* = (*F*_o_^2^ + 2*F*_c_^2^)/3
Δρ_max_, Δρ_min_ (e Å^−3^)	2.22, −4.45

Additional computer programs: *OLEX2* (v. 1.5; Dolomanov *et al.*, 2009 ▸).

**Table 2:** Distance of S atoms in the superstructure to those in the average structure

Atoms	*d* (Å)	Atoms	*d* (Å)
S1*A*/S1*B*	0.062	S8*A*/S8*B*	0.105
S2*A*/S2*B*	0.028	S9*A*/S9*B*	0.213
S3*A*/S3*B*	0.028	S10*A*/S10*B*	0.193
S4*A*/S4*B*	0.074	S11*A*/S11*B*	0.150
S5*A*/S5*B*	0.094	S12*A*/S12*B*	0.261
S6*A*/S6*B*	0.051	S13*A*/S13*B*	0.135
S7*A*/S7*B*	0.200	S14*A*/S14*B*	0.198

**Table 3:** Distance of *M* atoms in the superstructure to those in the average structure, whereby sites with occupancies < ½ were ignored Occupancies of the 0 < *x* < ½ and *x* < ½ < 1 sites are listed in the second and third column, respectively.

Sites	*d* (Å)	occ. 0 < *x* < ½	occ. ½ < *x* < 1
*M*1*A*/*M*1*B*	0.031	Pb	Pb
*M*2*A*/*M*2*B*	0.026	Pb	Pb
*M*3*A*/*M*3*B*	0.061	Pb	Pb
*M*4*A*/*M*4*B*	0.245	76% Pb, 24% Sb	90% Ag, 10% Cu
*M*5*A*/*M*5*B*	0.079	95% Sb, 5% Pb	97% Sb, 3% Pb
*M*6*A*/*M*6*B*	0.103	98% Sb, 2% Pb	73% Pb, 27% Sb
*M*7*A*/*M*7*B*	0.032	Pb	Pb
*M*8*A*/*M*8*B*	0.109	Sb	Sb
*M*9*A*/*M*9*B*	0.177	Sb	Sb
*M*10*A*/*M*10*B*	0.109	Sb	Sb
*M*11*A*/*M*11*B*	0.136	93% Sb, 7% Pb	97% Sb, 3% Pb
*M*12*A*/*M*12*B*	0.091	Ag	Ag

## References

[bb60] Dolomanov, O. V., Bourhis, L. J., Gildea, R. J., Howard, J. A. K. & Puschmann, H. (2009). *J. Appl. Cryst.* **42**, 339–341.

[bb1] Dornberger-Schiff, K. & Grell-Niemann, H. (1961). *Acta Cryst.* **14**, 167–177.

[bb2] Ďurovič, S. (1979). *Krist. Techn.* **14**, 1047–1053.

[bb3] Ferraris, G., Makovicky, E. & Merlino, S. (2008). *Crystallography of Modular Materials*, vol. 15 of *IUCr Monographs on Crystallography.* Oxford: Oxford University Press.

[bb4] Grell, H. (1984). *Acta Cryst.* A**40**, 95–99.

[bb5] Laufek, F., Pažout, R. & Makovicky, E. (2007). *Eur. J. Mineral.* **19**, 557–566.

[bb6] Makovicky, E. (1985). *Z. Kristallogr.* **173**, 1–23.

[bb7] Makovicky, E. & Olsen, P. N. (2015). *Can. Mineral.* **53**, 879–884.

[bb8] Moëlo, Y., Makovicky, M., Mozgova, N. N., Jambor, J. L., Cook, N., Pring, A., Paar, W., Nickel, E. H., Graeser, S., Karup-Møller, S., Balic-Žunic, T., Mumme, W. G., Vurro, F. & Topa, D. (2008). *Eur. J. Mineral.* **20**, 7–62.

[bb9] Rigaku Oxford Diffraction (2023). *CrysAlisPro* software system, version 1.171.42.85. Rigaku Corporation, Wrocław, Poland.

[bb10] Robinson, S. C. (1949). *Am. Mineral.* **34**, 398–402.

[bb11] Shannon, E. V. (1921). *Am. Mineral.* **6**, 82–83.

[bb12] Sheldrick, G. M. (2015*a*). *Acta Cryst.* A**71**, 3–8.

[bb13] Sheldrick, G. M. (2015*b*). *Acta Cryst.* C**71**, 3–8.

[bb14] Smaalen, S. van (2007). *Incommensurate Crystallography*, Vol. 21 of *IUCr Monographs on Crystallography.* Oxford: Oxford University Press.

[bb15] Stöger, B. & Weil, M. (2013). *Mineral. Petrol.* **107**, 253–263.

